# Estimating social bias in data sharing behaviours: an open science experiment

**DOI:** 10.1038/s41597-023-02129-8

**Published:** 2023-04-21

**Authors:** Claudia Acciai, Jesper W. Schneider, Mathias W. Nielsen

**Affiliations:** 1 Department of Sociology, University of Copenhagen, Øster Farimagsgade 5, 1353 Copenhagen, Denmark; 2 Danish Centre for Studies in Research and Research Policy, Department of Political Science, Aarhus University, Bartholins Allé 7, 8000 Aarhus C, Denmark

**Keywords:** Scientific community, Social sciences

## Abstract

Open data sharing is critical for scientific progress. Yet, many authors refrain from sharing scientific data, even when they have promised to do so. Through a preregistered, randomized audit experiment (N = 1,634), we tested possible ethnic, gender and status-related bias in scientists’ data-sharing willingness. 814 (54%) authors of papers where data were indicated to be ‘available upon request’ responded to our data requests, and 226 (14%) either shared or indicated willingness to share all or some data. While our preregistered hypotheses regarding bias in data-sharing willingness were not confirmed, we observed systematically lower response rates for data requests made by putatively Chinese treatments compared to putatively Anglo-Saxon treatments. Further analysis indicated a theoretically plausible heterogeneity in the causal effect of ethnicity on data-sharing. In interaction analyses, we found indications of lower responsiveness and data-sharing willingness towards male but not female data requestors with Chinese names. These disparities, which likely arise from stereotypic beliefs about male Chinese requestors’ trustworthiness and deservingness, impede scientific progress by preventing the free circulation of knowledge.

## Introduction

Scientific discoveries are made through cumulative and collective efforts, ideally based on full and open communication. For science to work, published claims must be subject to organized skepticism^1^. Yet, science’s ethos of rigorous, structured scrutiny is contingent on data sharing. Lack of data prevents results from being reexamined with new techniques, and samples from being pooled for meta-analysis^2,3^. This ultimately hinders the cumulative knowledge-building that drives scientific progress^4^.

Open data improves the credibility of scientific claims, and while journal editors increasingly acknowledge the importance of disclosing data^5,6^, many authors refrain from sharing their data, even when they have promised to do so^7–13^.

Previous research has focused on the supply-side determinants of data-sharing. Surveys find that scientists’ decisions to share data depend on (i) contextual factors such as journal requirements, funding incentives and disciplinary competition, (ii) individual factors such as perceived risks of data misuse, lost publication opportunities, and efforts associated with making data available, and (iii) demographic factors such as experience level, tenure-status and gender^7,14–19^.

Much less attention is given to the demand-side issues of data sharing. Ideally, everyone, irrespective of background, should be able to contribute to science^1^. As such, data access should not differ depending on who is asking for the data. Yet, research indicates persistent gender, ethnic and status-related bias in science^20–27^ that likely also affects data-sharing practices.

Social bias in data-sharing may arise from scientists’ stereotypic beliefs about data requestors. According to status characteristics theory, nationality, ethnicity, gender and institution prestige are diffuse cues that, when salient, may influence scientists’ impressions of requestors’ trustworthiness, competence or deservingness^28,29^. Such status cues are more likely to guide people’s judgments in ambiguous situations, where information is scarce^30,31^. Further, status cues may be critical for data sharing, as knowledge transfer is generally more likely in high-trust situations, here including the potential data sharer’s trust in the requestor’s competences and intentions^32,33^.

We conducted a pre-registered (https://osf.io/378qd), randomized audit experiment to examine possible ethnic, gender and status bias in data sharing. We requested data from authors of papers published in widely recognized journals that commit to the FAIR data principles (*the Proceedings of the National Academy of Sciences* [PNAS] and *Nature*-portfolio outlets), where data were indicated to be available upon request. We varied the identity of the fictitious data requestor on four factors: (i) country of residence (China vs. United States [US]), (ii) institution prestige (high status vs. lower status university), (iii) ethnicity (putatively Chinese vs. putatively Anglo-Saxon), and (iv) gender (masculine-coded vs. feminine-coded name).

Motivated by evidence of gender, ethnic and status bias in trust games^34,35^, in correspondence tests of employers, legislators, educators and citizens^27,36–39^, and in field- and survey experiments of peer evaluation^22,40,41^, we hypothesized that scientists would be less willing to share data, when a requestor (i) was from China (compared to the US); (ii) was affiliated with a lower-status university (compared to a higher status university); (iii) had a Chinese-sounding name (compared to a typical Anglo-Saxon name); and (iv) had a feminine-coded name (compared to a masculine-coded name).

In addition to gender and institution status, which have previously been covered in correspondence tests of scientists^42,43^, we were interested in the specific disadvantages facing researchers with Chinese names and university affiliations. China is currently the world’s largest producer of scientific outputs^44^ and Chinese expatriates by far outnumber any other group of foreign graduate students at US universities^45,46^. Considering these figures, a study of the possible bias facing Chinese nationals and descendants in a globalized science system seems timely.

## Materials and Methods

### Sampling

Our data collection included four steps, summarized in Figure S1. The experimental population consisted of authors of scientific papers published in PNAS and Nature-portfolio journals (between 2017 and 2021), where data were indicated to be ‘available upon request’. We queried journal websites to identify all peer-reviewed papers that included the text-string “available upon request”. This resulted in an initial sample of 6,798 papers. C.A. and M.W.N. manually checked and coded the data availability section of each paper to identify cases where data were unambiguously made available upon request (Table S1). If the primary author contact listed in a data statement occurred multiple times in our sample (due to >1 publications), we only included the author’s most recent publication. We removed all retracted papers and sources with corrections. We matched the authors listed as primary data contacts in the papers with bibliographic metadata from Clarivate’s Web of Science (WoS) to retrieve up-to-date information on emails and affiliations (Figure S2). Our final sample consists of 1,826 author-paper pairs. Due to bounced emails and authors withdrawing from the study after receiving a debriefing statement (in total 78 authors decided to withdraw), our analysis sample is further reduced to 1,634 author-paper pairs. According to our registered power analysis, this sample size should be sufficient for detecting a small effect size of Cohen’s f^2^ = 0.02, with α = 0.01 and a power of 0.95.

### Procedures

Efforts to measure ethnic, gender and status bias are complicated by observer effects and issues of social desirability^47^. Audit studies can mitigate such biases by allowing the experimenter to estimate participants’ responses to the treatment in a realistic setting^47–49^.

Our unpaired audit study randomized participants across twelve treatment conditions (Figure S3). Given our four treatments, a 4 × 4 factorial design would be the typical set-up for our study. Yet, to keep the treatments realistic, we decided not to include a putatively Anglo-Saxon (male or female) treatment associated with a (higher or lower status) Chinese University (during the COVID-19 pandemic, the number of international students in China decreased enormously). Thus, we adopted 12, instead of 16, treatment conditions. Previous research indicates that data-sharing practices differ depending on the author’s gender and disciplinary field^11,13,50^. Hence, we block-randomized the sample population according to scientific field and gender. Inspired by previous audits on discrimination in science^42,43^, our data-sharing requests were emailed from fictitious “about-to-become” PhD students. We created Gmail accounts for all of the gender-ethnicity combinations (in total 4 email addresses). In the emails, the fictitious data requestors asked the participants to share data related to a specific publication (Figure S4). If participants did not respond to our initial email, we sent out a follow-up request after one week. Data collection was completed two weeks after the follow-up request. All data were collected in April and May 2022. Email correspondences were managed through YAMM (https://yamm.com/), a private email service provider (google sheet add-on for Gmail and Google Workspace users). This tool allowed us to track the email delivery metrics and provided information on whether an email had been received (or bounced) and opened (or left unopened).

Our sampling and analysis plan was preregistered at the Open Science Framework. We have followed all steps presented in the registered plan, with one minor deviation. In the preregistration we planned to run two linear probability models with cluster-robust standard errors at the field level, while in the results section, we report the outcomes of these models without the cluster-robust standard errors. Cluster robust standard errors proved unsuitable given the low number of clusters and because randomized treatments were assigned at the individual level as opposed to the group-level^51^.

### Manipulations

Our treatment conditions varied on the following four factors: gender, ethnicity, country of residence and institutional affiliation. We used first and last names to signal the fictitious requestors’ gender and ethnicity (Figure S4). Emails from putatively Chinese treatments included a masculine- or feminine-coded Anglo-Saxon middle name in a parenthesis to signal the requestor’s gender (e.g., ‘Yadan (Cecilia) Xing’). This is a widely used name-practice among transnational Chinese students^52^. We created four email addresses for our treatments. All accounts were opened and used for at least 90 days before the experiment started. Warm-up activities of email domains (e.g., sending small volumes of emails and slowly increasing) helped build a positive sender reputation for the accounts. Such standard email activity from the newly created account can reduce the risk of an account being labeled as a fake, hence reducing the email’s likelihood of being filtered as spam. To select relevant names, we relied on a historical dataset of Olympic athletes^53^. We limited our focus to Chinese and American athletes participating in the Olympic Games between 1932 and 2016. For each country treatment (US and China), we randomly selected separate first and last names until finding appropriate combinations. For the putatively Anglo-Saxon requestor conditions, we used the following names: Jeffrey Killion and Hilary Witmer. For the putatively Chinese requestor conditions, we used the following names: 张嘉实 (Jia Shi Zhang) and 邢雅丹 (Yadan Xing). We used the R package “rethnicity”^54^ to ensure that the selected Anglo-Saxon first names were typical Anglo-Saxon names and that the selected Chinese first names were well-known Asian names. In addition, we manually verified the rethnicity estimates by looking up the first names on Linkedin. To ensure that participants perceived the Chinese names as Chinese, we wrote the signature names (at the bottom of each email) in both English letters and Chinese characters (‘Yadan (Cecilia) Xing | 邢雅丹’ and ‘Jiashi (Wilson) Zhang | 张嘉实’) (see also Figure S4). According to the US Census of Frequently Occurring Surnames^55^, 96% of individuals with the surname “Witmer” identified as white, 87% of individuals with the surname Killion identified as white, 97% of individuals with the surname Xing identified as Asian, while 98% with the surname Zhang identified as Asian. Previous research suggests that some race and ethnicity-based manipulations also include class primes^56^. In our design, we followed Block *et al*.^36^ and attempted to reduce the potential effect of socio-economic status by holding occupation constant. Specifically, all participants received an email from an ‘about to become’ Ph.D. student at a Chinese or US research institution.

Emails from putatively Chinese treatments (located in the US or China) that targeted authors outside of China were written in English. Emails from Chinese nationals that targeted authors located in China were written in Mandarin. We used four international university rankings to identify appropriate university affiliations that varied in institution status (high status [Carnegie Mellon and Zhejiang University] vs. lower status [Baylor University and Chongqing University) (SI *Appendix*, Table S2). This manipulation also signaled the fictitious requestors’ country of residence (China or the US). Appropriate university affiliations were identified using a combination of different university rankings (Times Higher Education, Shanghai national and international rankings, QS ranking, and the PP-top 10% indicator from the Leiden Ranking). To select high status affiliations, we focused on universities that scored consistently high across the rankings. When selecting lower-status affiliations, we focused on universities that scored consistently low across rankings. We decided to exclude top-ranked (top 10%) and bottom-ranked universities (bottom 10%) to lower the likelihood that participants would discern the purpose of the experiment. In addition, we restricted our selection to universities with multiple faculties and active Ph.D. programs within each faculty.

### Measures

We measured the experimental treatments using four dichotomous variables: country (US = 0, China = 1), ethnicity (Anglo-Saxon name = 0, Chinese name = 1), institution status (high status university = 0, lower status university = 1), and gender (masculine coded name = 0 and feminine coded name = 1). In addition, all statistical models included five dichotomous variables to adjust for scientific field and publication outlet (Table S3).

Our preregistered dependent variable is a binary measure of data-sharing willingness. This measure is based on a systematic coding of participants’ email responses (respondents did not share data nor indicate willingness to share data = 0, respondents shared data or indicated willingness to share data = 1). Two authors systematically coded all responses. The codebook (Table S4) was first tested by coding 10% of the sample. The pilot phase was repeated, and the codebook was further adjusted, until coding reliability measures reached a satisfactory level (Kappa coefficient ≥ 0.8). In the manual coding, the coders were blinded to the treatments.

As a second outcome (not registered), we measured whether participants responded to our data requests or not (non-response = 0, response = 1). This outcome measure is widely used in previous email-based correspondence tests^57^. As opposed to data-sharing willingness, our measure of email responses did not involve any coding of textual content and can thus be viewed as a more objective indicator of bias in data sharing.

**Fig. 1:**
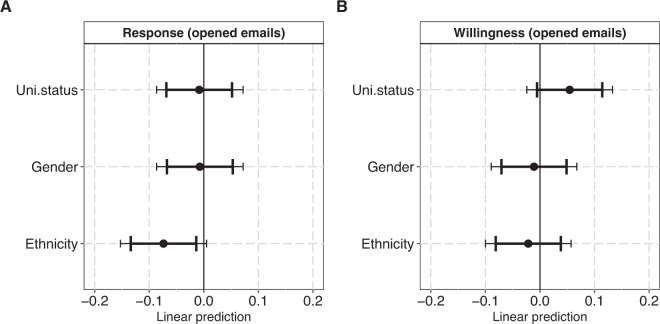
Plots of coefficients from two linear probability models with “Response” (Panel A) and “Willingness” (Panel B) as outcomes. The sample (N: 770) covers all participants (that have opened the treatment email[s]) exposed to treatments with US affiliations. The panels plot the fixed coefficients for the three main predictors University status (high = 0, low = 1), Gender (masculine-coded name = 0 feminine-coded name = 1) and Ethnicity (typical Anglo-Saxon name = 0, Chinese-sounding name = 1). Error bars represent 95% and 99%. Both models adjust for scientific field and publisher. For model specifications, see Supplementary Tables S6, S7.

In accordance with our registered analysis plan, we measured and reported the dependent variables in two ways. In one case, we excluded all unopened emails from the analysis. In the results section, we refer to this sample as the sample of “opened emails”. In the other case, we included unopened emails and coded them as indicating unwillingness to share data or non-responses. In the results section, we refer to this sample as the “full sample”. Given that all participants in the sample of opened emails have been directly exposed to a treatment, we would expect the treatment effects to be larger for this sample. In contrast, given that some participants in the full sample were not directly exposed to a treatment, we would expect the noise-to-signal ratio to be larger and the treatment effects to be smaller for this sample. Thus, while the sample of opened emails gets us closer to the direct effect of the treatments, the full sample gives us a better sense of the real-world disadvantages associated with a given treatment. Due to a minor error in our email management, responses to one of the treatment emails were associated with an alias during the first wave of data collection. Specifically, participant responses to emails sent from Yadan Xing were forwarded to a different alias address with a similar manipulation condition. Only two participants noticed this issue in their responses, but nonetheless positively engaged with our request. The rest of the respondents exposed to this error either responded to the alias email while addressing their message to Yadan (e.g., “Dear Yadan”) or responded directly to the correct email manipulation. Based on this evidence, we find it reasonable to assume that the vast majority of respondents exposed to the error either did not notice the issue or perceived it to be irrelevant. This mistake was corrected in the follow-up email addressed to all recipients who did not respond to the first email. Table S5 presents the response rates across treatments for the first and the second wave. The treatment including the alias mistake (Yadan Xing) had the highest response rate of the four treatment emails after wave one. Note also that our main findings concerning a bias in responsiveness and data-sharing willingness (Figs. 1,4) pertain to the male Chinese treatment (Jiashi Zhang) and not the female Chinese treatment (Yadan Xing).

### Statistical analyses

**Fig. 2:**
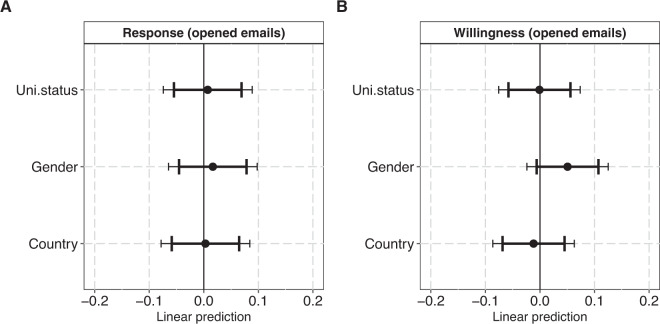
Plots of coefficients from two linear probability models with “Response” (Panel A) and “Willingness” (Panel B) as outcomes. The sample (N: 802) covers all participants (that have opened the treatment email[s]) exposed to treatments with Chinese-sounding names located in the US and China. The panels plot the fixed coefficients for the three main predictors University status (high = 0, low = 1), Gender (masculine-coded name = 0 feminine-coded name = 1) and Country (US university = 0, Chinese university = 1). Error bars represent 95% and 99%. Both models adjust for scientific field and publisher. For model specifications, see Supplementary Tables S10, S11.

Given that no participants in our study were exposed to the putatively Anglo-Saxon treatments located in China, we estimated two groups of linear probability models. In one group, we estimated the direct effects of gender, ethnicity and institution prestige on data sharing and email responses among participants exposed to treatments affiliated with US universities (Fig. 1 and S5). In another group, we estimated the direct effects of gender, country-location, and institution prestige on data-sharing willingness among putatively Chinese treatments located in the US and China (Fig. 2 and S6). Despite the unidirectional nature of our hypotheses, we report all outcomes with two-directional 95% and 99% confidence intervals.

All analyses were conducted in R^58^, and we used the estimator package^59^ to perform the linear probability models.

### Ethics

Since the social biases examined in our study may operate subconsciously, a pre-experimental consent process could damage the validity of the experiment. For this reason, we decided to operate with ex-post consent and information disclosure. Our study was approved by the Ethics Review Board at the Department of Sociology, University of Copenhagen (UCPH-SAMF-SOC-2022-03). At the end of the experiment, a debriefing email was sent to all participants (non-respondents as well as respondents). In this debriefing, we explained the general purpose of the study, its experimental manipulations, the potential risks and benefits to participants, and the principles of data management and anonymization. Moreover, we informed participants about their right to withdraw from the study without penalty. In total, 78 authors (5%) decided to withdraw from our study after receiving the debriefing statement.

## Results

Eight-hundred-and-eighty-four scientists responded to our data requests, and 226 either shared or indicated willingness to share all or some of their data. This corresponds to 54% (884 of 1634) and 14% (226 of 1634) of the full sample, and 75% (884 of 1179) and 20% (226 of 1179) of the sample of opened emails.

In Fig. 1, we estimate how institution prestige, gender and ethnicity influence participants’ responsiveness and data-sharing willingness in the sub-sample (that opened emails) exposed to treatments with US affiliations. As shown in the figure (Panel A), neither university status nor gender affected participants’ likelihood of responding to data requests from treatments with US affiliations. Yet, treatments with Chinese-sounding names were 7 percentage points less likely to receive a response than treatments with putatively Anglo-Saxon names (β = −0.07, 95% CI: −0.13:−0.01; 99% CI: −0.15:0.01). This corresponds to an odds ratio of 0.66, or 34% lower odds of obtaining a response for the treatments with Chinese-sounding names compared to putatively Anglo-Saxon treatments (Table S16). Results are similar, though associated with larger uncertainties, when estimates are based on the full sample as opposed to the sample of opened emails (Figure S5).

Our pre-registered analysis of ethnic, gender and status-related bias in scientists’ willingness to share data with treatments located in the US proved inconclusive in both the full sample (Figure S5, Panel B) and in the sample of individuals that opened emails (Fig. 1, Panel B). In conflict with one of our hypotheses, participants seemed slightly more willing to share data, when the request came from a lower status US institution (Baylor University) compared to a higher status US institution (Carnegie Mellon), but the confidence intervals for this effect all include zero (β = 0.05, 95% CI: −0.01:0.11; 99% CI: −0.02:0.13).

In Fig. 2, we estimate how university status, gender and country matter for participants’ responsiveness and data-sharing willingness in the sub-sample exposed to treatments with Chinese-sounding names, located in China vs. the US. As shown in Fig. 2 (Panel A), we do not find any association between the treatment conditions and participants’ responsiveness to data-requests in this subsample. All coefficients are close to zero, indicating no discernible effects. Similarly, for data-sharing willingness (Fig. 2, Panel B), results are weak and inconclusive. The effects of university status and country on data-sharing willingness are close to zero, albeit women, not men (contrasting our pre-registered hypothesis), are met with slightly higher data sharing willingness in this subsample. Yet, the confidence intervals for this gender effect include zero (β = 0.05, 95% CI: −0.01:0.11; 99% CI: −0.02:0.13). Again, the results are similar, when models are based on the full sample as opposed to the sample of opened emails (Figure S6).

**Fig. 3:**
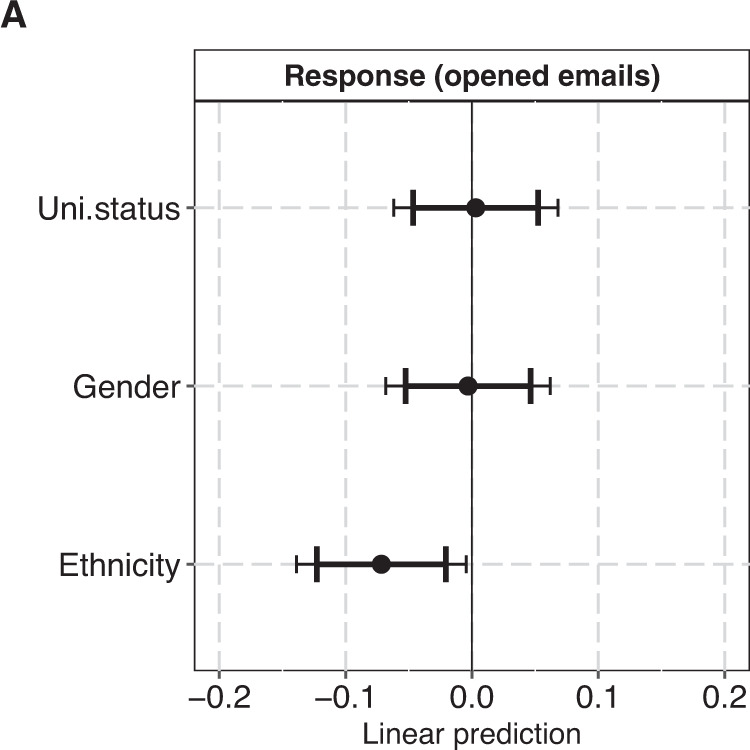
Plots of coefficients from a linear probability model with “Response” as outcome. The sample (N: 1179) covers all participants that have opened the treatment email(s). The panel plots the fixed coefficients for the three main predictors University status (high = 0, low = 1), Gender (masculine-coded name = 0 feminine-coded name = 1) and Ethnicity (Typical Anglo-Saxon name = 0, Chinese-sounding name). Error bars represent 95% and 95%. The model adjusts for scientific field and publisher. For model specifications, see Supplementary Table S14.

In Fig. 3, we explore if the ethnicity bias indicated in Fig. 1 (Panel A) is specific to the US treatments with Chinese-sounding names, or whether it affects putatively Chinese data requestors more generally. In this analysis, which covers both the US-located and China-located treatments, we obtain results comparable to those reported in Fig. 1 (Panel A). The effects for university status and gender remain inconclusive, but treatments with Chinese-sounding names have a 7 percentage points lower likelihood of receiving a response than treatments with typically Anglo-Saxon names (β = -0.07, 95% CI: -0.12:-0.02; 99% CI: -0.14:-0.00). This corresponds to an odds ratio of 0.67, or 33% lower odds of response for putatively Chinese treatments compared to putatively Anglo-Saxon treatments (Table S20). This estimated effect is smaller and associated with larger uncertainties when the analysis is based on the full sample compared to the sample of opened emails (Figure S7).

**Fig. 4:**
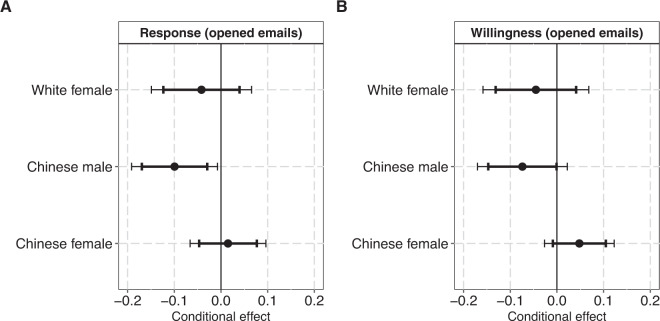
Conditional coefficients derived from interactions between Ethnicity and Gender in two linear probability models with Response (Panel A) and Willingness (Panel B) as outcomes. The sample (N: 1179) covers all participants who have opened the treatment email(s). The panels plot conditional coefficients of the ethnicity variable by treatment gender. The reference group is the putatively Anglo-Saxon male treatment. Errors bars represent 95% and 99% confidence intervals. For model specifications, see Supplementary Tables S22, S23.

Given the indication in Fig. 2 (Panel B) that data-sharing willingness is lower for men with Chinese names compared to women with Chinese names, we also estimated the conditional effects of ethnicity on data-sharing behaviors for male and female treatments. To secure the highest possible statistical power, we ran interaction-analyses on the combined samples of US-located and China-located treatments. Figure 4 plots the conditional coefficients from four interactions between Ethnicity and Gender in the sample of authors that opened emails. Male treatments with Chinese-sounding names (compared to male treatments with putatively Anglo-Saxon names) face consistent disadvantages both with respect to responsiveness (β = −0.10, 95% CI: −0.17:-0.03; 99% CI: −0.19:-0.01) and willingness (β = −0.07 95% CI: −0.15:-0.00; 99% CI: -0.17:0.02) when requesting data, while this is not the case for female treatments with Chinese-sounding names. We obtain comparable results when the conditional coefficients are estimated based on the full sample (Figure S8).

## Discussion

Previous research on social bias in data sharing is scarce. Several studies document low response rates and low data-sharing willingness among scientists who agreed to make their data available upon request^7,9,11,12,15^. Yet, demand-side determinants of data sharing remain largely unexamined.

In a small-scale field experiment (N = 200), Krawczyk and Reuben^60^ tested whether economists’ willingness to share supplementary materials differed depending on the prestige of the requestor’s university and position level (Columbia University vs. University of Warsaw) and found negligible effects.

In our audit experiment, which draws on a larger multi-disciplinary sample of participants, none of the four preregistered hypotheses predicting national, ethnic, gender and institutional prestige bias in data-sharing willingness were supported.

Yet, tests for differences in scientists’ responsiveness to data requests (our most objective measure of disparities in data-sharing) indicated lower response rates for Chinese treatments compared to Anglo-Saxon treatments. This may indicate that ethnic bias is more likely to occur at the initial stage of a (potential) data exchange when scientists make rapid and unreflective judgments on whether to engage with a requestor or not. Indeed, previous research into ethnic bias in pro-sociality also emphasizes the role of implicit attitudes (activated quickly and spontaneously) in discriminatory behaviors^61,62^.

Contrary to our expectations, scientists exposed to US-located treatments seemed slightly less willing to share data with requestors from prestigious universities compared to requestors from lower status universities (although the 95% confidence bounds spanned zero for this estimate). One possible explanation for this may be that the perceived career risks associated with data sharing (in terms of lost publication opportunities and lowered competitive advantages), on average, are higher, when requests are made from prestigious US universities compared to lower-status US universities or Chinese universities. Indeed, previous research finds that scientists’ data-sharing willingness tends to be lower when perceived competition is high^7,50,63,64^. Such risks and concerns could potentially be reduced through the use of data licensing on public repositories like OSF.

Importantly, the negative prestige effect was only salient for scientists exposed to treatments from US universities. This may be because the participants in our sample, which primarily reside in Europe or North America (Table S26), are more familiar with the prestige hierarchy of US institutions and less knowledgeable about the relative standing of Chinese institutions.

Also contrary to our predictions, Chinese treatments with feminine-coded names were met with slightly higher data-sharing willingness than Chinese treatments with masculine-coded names (although the 95% confidence bounds spanned zero for this estimate). Importantly, this finding reflects an underlying pattern of male-specific ethnic discrimination. Conditional effects derived from interaction analyses suggested a clear bias in responsiveness and data-sharing willingness against male Chinese treatments, while results for female Chinese treatments were inconclusive.

Given the “double-burden hypothesis” in intersectional theory^65^, which states that minority women are most likely to face discrimination, these findings may seem counter-intuitive. Nevertheless, they have some bearing in previous studies on trust and discrimination. Indeed, evidence suggests that women are stereotypically seen as more trustworthy than men^66^, and studies on helping behavior also indicate a greater taste for helping women than men in various social situations^67–70^. Further, while field experiments have generally neglected intersectional perspectives on ethnic and gender discrimination^71^, studies that *do* cover this aspect typically find that minority males are subject to larger ethnic penalties than minority females in job-markets, housing markets, and sharing economies^72–79^. Building on research on gender and nationality stereotypes^80^, Arai and colleagues theorize that when stereotypes against specific ethnic groups are negative, they are more likely to disadvantage men than women, because it is men who are primarily presumed to embody these stereotypes^72^. Additional research is required to determine the root causes of the observed bias towards Chinese men. However, we hypothesize that it likely arises from stereotypic beliefs about the group’s trustworthiness and deservingness in data exchange relationships. Such beliefs may have been particularly salient during 2022, when we collected our data, due to recurring discussions about China’s alleged intellectual property theft in the US and Europe^81^, but also in the wake of COVID-19, where prejudice and discriminatory intent against Asians aggravated^82,83^.

While our field-experiment has clear advantages over survey-based approaches to measuring data-sharing behaviors, it is not without limitations. Our data requests were sent from Gmail accounts, and this may have increased the likelihood that emails ended up in the recipients’ spam filters. Further, some recipients may have found our data requests more suspicious than they would have, if the same emails were sent via institutional accounts. We have attempted to account for this issue by tracking opened and unopened emails throughout the study-period and by reporting results for samples that include and exclude unopened emails (SI *Appendix*).

Compared to previous correspondence studies, where data-requests were made from institutional email accounts, the response rate in our study is quite high. Participants ignored 49% of the requests made by Tedersoo and colleagues^11^ concerning data for recent papers in *Nature* and *Science*; while 86% of the data requests made by Gabelica and colleagues to authors publishing in BioMed Central journals were also ignored^12^. In comparison 54% of scientists in our sample responded to the data requests. This suggests that the drawbacks of using Gmail compared to an institutional account have been small.

Another limitation of our study design concerns the generic nature of our data request, which may have increased the level of suspicion among some recipients. After the study was completed, a few authors approached us with concerns that the email request lacked detail and was not tailored to the specific practices of their discipline, which made them hesitant to respond. While we acknowledge this critique, experiments like ours will always be subject to trade-offs between ecological validity and treatment bias. In this case, we decided to keep the emails generic to hold constant all other components than our manipulations.

A fourth limitation concerns our sampling strategy. Because we only targeted authors of papers in Nature portfolio journals and PNAS, our results are limited to scientists publishing in these journals. In the future, researchers should examine whether data sharing behaviors differ for authors publishing in journals that are less committed to open science and the FAIR data-sharing principles. Finally, given that none of our four preregistered hypotheses were directly confirmed, our results concerning gender-specific ethnic discrimination in data sharing can only be seen as suggestive.

Despite these limitations, our paper offers important new insights on scientific data-sharing practices in science. Compared to unregistered experiments, our preregistered analysis has the advantage of providing a clear record of what ideas our study was designed to evaluate, how we planned to examine them, and how our most notable finding of ethnic bias in data-sharing relate to these ideas^84^. Put differently, the preregistered analysis plan has limited our degrees of freedom as researchers and thereby increased the validity and reliability of our study.

Our paper has important implications for open science policies. Despite clearly indicating intent to make their data available upon request, only around half of the targeted authors responded to our data requests, and only 14% indicated willingness to share all, or some, of their data. While some participants may have had good reasons not to share, this behavior conflicts with the FAIR principles adopted by PNAS and Nature portfolio journals, hence demonstrating the drawbacks of enabling researchers to make data available upon request. Our study further complicates this issue by exposing potential inequalities in who can benefit from data-sharing, when disclosure decisions are left to the discretion of individual scientists^7^. In accordance with previous work, our study shows that data requests often require more than trivial efforts from the side of the requestor. These efforts could be reduced if funders and publishers required authors to release all relevant data, whenever possible^11,12^.

Unfortunately, the reality is that most journals do not incentivize data sharing. In a review of editorial policies for 318 biomedical journals^5^, only 12% explicitly required data sharing as a condition for publication, 9% required data sharing without stating it as a condition for publication, while around one third of the journal sample did not mention data-sharing at all. Under such conditions, we expect that “data availability upon request” will remain a widespread practice in many disciplines.

Importantly, disclosure is sometimes also challenged by practical issues (e.g., data size and propriety rights) or ethical issues (e.g., sensitive information on human subjects), and publishers could do more to help mitigate these challenges. From our experience, it seems that many authors that cannot share their data for practical or ethical reasons currently opt to indicate data availability upon request to circumvent a journal’s data-sharing requirements. Assistance from the side of publishers in providing the necessary storage space or easy-to-use methods for making synthetic datasets for sensitive populations, could help mitigate these problems^85^.

In summary, our field experiment extends on research about scientists’ compliance with open data principles by indicating that sharing behaviors may differ depending on who is asking for the data. These disparities, which likely arise from stereotypic beliefs about specific requestors’ trustworthiness and deservingness, hamper the core principle of mutual accountability in science and impede scientific progress, by preventing the free circulation of knowledge.

## Supplementary information

Supplementary Information

## Data Availability

Data available at this link: osf.io/kzrc4^86^

## Code availability

Code can be accessed at this link: osf.io/kzrc4
