# Supplementary material for: Estimating social bias in data sharing behaviours: an open science experiment
Source: Sci Data. 2023 Apr 21;10:233. doi: 10.1038/s41597-023-02129-8 (PMC10120507; doi:10.1038/s41597-023-02129-8)
Supplement: Supplementary file 1 — Supplementary Information [file 41597_2023_2129_MOESM1_ESM.docx]

**Supplementary Information**

**Estimating social bias in data sharing behaviours: an open science experiment**

Claudia Acciai*, Jesper W. Schneider, Mathias W. Nielsen*

*Corresponding authors: [cla@soc.ku.dk](mailto:cla@soc.ku.dk), [mwn@soc.ku.dk](mailto:mwn@soc.ku.dk)

**Table of Contents**

**Figure S1** Core steps in data collection.

**Figure S2** Sample construction

**Figure S3** The twelve experimental treatment conditions.

**Figure S4** Contact email form.

**Figure S5** Plots of coefficients from two linear probability models

**Figure S6** Plots of coefficients from two linear probability

**Figure S7** Plots of coefficients from a linear probability model with “Response” as outcome.

**Figure S8** Conditional coefficients of ethnicity by gender derived from interactions in two linear probability models

**Table S1** Codebook for identifying research papers where data were available upon request.

**Table S2** University affiliations (Institution status) comparison across rankings.

**Table S3** Variable specifications.

**Table. S4** Codebook for inferring data-sharing willingness.

**Table S5** Response rate first and second round of emails (initial contact and reminder)**.**

**Table S6** LPMs presented in Figure 1 (Panel A).

**Table S7** LPMs presented in Figure 1 (Panel B).

**Table S8** LPMs presented in Figure S4 (Panel A).

**Table S9** LPMs presented in Figure S4 (Panel B).

**Table S10** LPMs presented in Figure 2 (Panel A).

**Table S11** LPMs presented in Figure 2 (Panel B).

**Table S12** LPMs presented in Figure S5 (Panel A).

**Table S13** LPMs presented in Figure S5 (Panel B).

**Table S14** LPMs presented in Figure 3 (Panel A).

**Table S15** LPMs presented in Figure S6.

**Table S16** Logistic regression model corresponding to LPMs presented in Figure 1 (Panel A).

**Table S17** Logistic regression model corresponding to LPMs presented in Figure 1 (Panel B).

**Table S18** Logistic regression model corresponding to LPMs presented in Figure S4 (Panel A).

**Table S19** Logistic regression model corresponding to LPMs presented in Figure S4 (Panel B).

**Table S20** Logistic regression model corresponding to LPMs presented in Figure 3.

**Table S21** Logistic regression model corresponding to LPMs presented in Figure S6.

**Table S22** LPMs presented in Figure 4 (Panel A).

**Table S23** LPMs presented in Figure 4 (Panel B).

**Table S24** LPMs presented in Figure S7 (Panel A).

**Table S25** LPMs presented in Figure S7 (Panel B).

**Table S26.** Geographical distribution of participants.

**Supplementary Figures**

**Figure S1** Core steps in data collection

**Figure S2** Sample construction. Attrition emails include bounced emails as well as participants who decided to withdraw from the study.

**Figure S3** The twelve experimental treatment conditions. We manipulated information about the requestors’: (i) country of residence, China vs. United States (marked with the vertical arrow “country”); (ii) institutional affiliations, high status vs. lower status university (marked with the vertical arrow “institution”); (iii) ethnicity, Chinese vs. typical Anglo-Saxon name (marked with the horizontal arrow “ethnicity”; (iv) gender, masculine-coded vs. feminine-coded name (marked with the horizontal arrows “gender”).

**Figure S4** Contact email form. Emails from Chinese data requestors to Chinese participants were written in Chinese. All other email correspondences were written in English.

**Figure S5** Plots of coefficients from two linear probability models with “Response” (Panel A) and “Willingness” (Panel B) as outcomes. The sample (N: 1092) covers all participants (that have *received* a treatment email) exposed to US-based treatments. The panels plot the fixed coefficients for the three main predictors University status (high=0, low=1), Gender (masculine-coded name=0 feminine-coded name=1) and Ethnicity (typical Anglo-Saxon name=0, Chinese-sounding name=1). Error bars represent 95% and 99% confidence intervals. Both models adjust for scientific field and publisher. For model specifications, see Supplementary Tables S8 and S9.

**Figure S6** Plots of coefficients from two linear probability models with “Response” (Panel A) and “Willingness” (Panel B) as outcomes. The sample (N: 1097) covers all participants (that have *received* a treatment email) exposed to treatments with Chinese-sounding names located in the US and China. The panels plot the fixed coefficients for the three main predictors University status (high=0, low=1), Gender (masculine-coded name=0 feminine-coded name=1) and Country (US university=0, Chinese university =1). Error bars represent 95% and 99% confidence intervals. Both models adjust for scientific field and publisher. For model specifications, see Supplementary Tables S12 and S13.

**
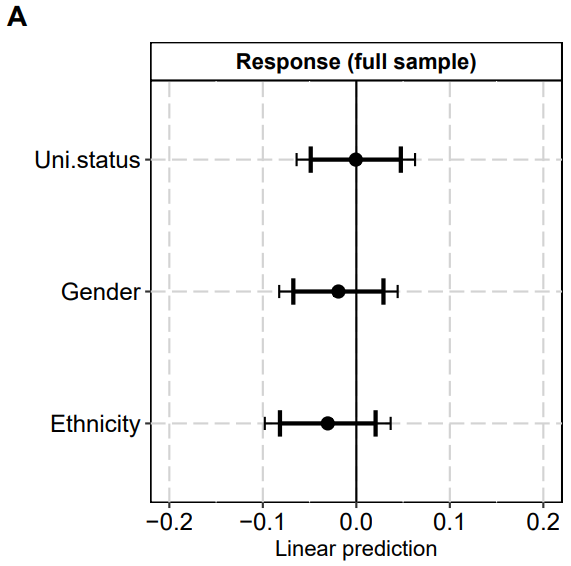
**

**Figure S7** Plots of coefficients from a linear probability model with “Response” as outcome. The sample (N: 1634) covers all participants that have received a treatment email. The panel plots the fixed coefficients for the three main predictors University status (high=0, low=1), Gender (masculine-coded name=0 feminine-coded name=1) and Ethnicity (Typical Anglo-Saxon name=0, Chinese-sounding name. Error bars represent 95% and 99% confidence intervals. The model adjusts for scientific field and publisher. For model specifications, see Supplementary Table S15.

**Figure S8** Conditional coefficients of ethnicity by gender derived from interactions in two linear probability models with (A) Willingness and (B) Response as outcomes. The sample (N: 1634) covers all participants (that have opened the treatment email(s)). The panels plot conditional coefficients of the ethnicity variable by treatment gender. The reference group is the putatively Anglo-Saxon male treatment. Errors bars represent 95% and 99% confidence intervals. For model specifications, see Supplementary Tables S24 and S25.

**Supplementary Tables**

**Table S1** Codebook for identifying research papers where data were available upon request.

| **CATGORIES** | **EXAMPLE** |
| --- | --- |
| **Yes (eligible)**  DATA IS AVAILABLE | **Data/Raw Data available upon request:**   - The raw datasets generated during and/or analyzed during the current study will be made available upon request to the corresponding author - The data (and reagents) that support the findings of this study are available from the corresponding author upon reasonable request - (XXX) data and all model-outputs including model and graphics codes will be available upon request - The authors agree to make materials, data and associated protocols promptly available to readers. - Primary data files are available upon request - Data including XX protocols and results are available upon request. - All data used in this analysis is from free, publicly available sources and is available upon request from the corresponding author. - The data that support this study are available from the corresponding author upon reasonable request. Raw XXX datasets are available upon request. Source data are provided with this paper. - All data used in this study is publicly available. All data or tools generated by this study will be made available upon request. - Some study data are available upon request |
|  | **Source data are provided, data (all the other data) are available upon request**   - Source data are provided as a Source Data file. Data are available upon request. - Source data are available, all the other data are available upon request. - All raw data are available from the corresponding authors on reasonable request. Source data are provided with this paper. - Data used to generate the above figures are available upon request from the authors (when authors only state that with no differentiation between data and source data). - Optimized geometries for the structures discussed in the text are available in the supplementary files in the online version of the paper. The XXX and XXX data that support the findings of this study are available from the authors upon request. - The XXX, XXX, and data set are available upon request from the corresponding author. XXX data are available via link. - All the source data for the main figures are included in the supplementary data. All data in this study are available from the corresponding authors upon request. We have deposited all XXXX to XXXX. Also, all XXX will be made available upon request. - Raw and processed XXXXX and XXX data, and XXXX video clips are currently being stored at the University of XXX. It is available upon request. |
| **No (not eligible)**  IF (SOME) DATA ARE AVAILABILE SOMEHERE | **No data availability statement provided**   - Data cannot be shared |
|  | **Data available upon request to a third party / produced by a third party**   - NGOs, public administration, hospital, private actor - Data available from official website (EC data, OECD, WHO etc.) - All raw real data used in this study were produced by the XX project - Contact listed not included among the authors of the paper. |
|  | **There are (some sort of) privacy restrictions, data (all the other data/ a portion of data) is available upon request**   - Due to Institutional Review Board restrictions, the data are not publicly available. - Data cannot be shared, but some parts of the data are available upon request. - All the original data which were available upon request were kept in the database management system within the (Institution of the authors) - Data for this paper are stored on our institutional storage cloud and will be freely available upon request, but due to institutional policy on cybersecurity, specific access links have to be generated upon request rather than persistent public links. The total data volume available is several terabytes in size. - IRB allowing them to share data but not upload them publicly - Data can be made available upon request through a data sharing contract - Data will be available upon request after the execution of a data use agreement - The data are not publicly available due to them containing proprietary information. However, the data that support the findings of this study are available upon request. Specific data transfer agreements may be required for each individual request. - Due to restrictions related to participant consent, all relevant data are available upon request. More information is available at the following - Data will be made available, upon permission from the host institution, as the data controller - Data used in this study have been collected in a clinical study and are subject to regulations of the Ethics Committee of the XXX that approved these studies. |
|  | **Some data has been deposited somewhere or in the supplementary information and/or some (all/raw data) is available upon request**   - All study data are included in the article and/or SI Appendix. All materials are available upon request to corresponding author. - Data in supplementary material, online repository - Data and scripts are openly available at “http” (or supplementary/additional info) are available upon request from the first author. - The authors declare that all other data supporting the findings of this study are available within the article, Supplementary Information files or available from the authors upon reasonable request - The data that support the findings of this study are available within the paper, Supplementary Information, or available from the corresponding author upon request. - The authors declare that all other data supporting the findings of this study are available within the article, Supplementary Information files are available from the authors upon reasonable request. - All relevant data are available from the authors. Raw data have been deposited within XXX repository - All data are available in the manuscript or the supplementary materials OR from the authors on request. - All data generated or analyzed during this study are included in this published article. All relevant data are also available upon request from the authors. - The datasets generated during and/or analyzed during the current study, and not submitted as supplementary material, are available from the corresponding author on reasonable request - Most of the data generated or analyzed during this study are included in this published article (and its Supplementary Information files). The rest of the raw data generated during and/or analyzed during the current study are available upon request from the corresponding authors. - The authors confirm that all data supporting this method are available within the article and its supplementary materials and with the authors and are available upon request. - When portion of data are shared between authors. For data XX contact X.X., for that XX contact. - All data reported in the text have been compiled in the figures. All materials generated in this study are available upon request. - All processed data will be made available upon request and in a repository - The XXXX data were derived from the XXXX and XXXX data sets, available from the data portal ( http….). The XXX emissions inventory data are downloaded from http.. The XXXX XXXX can be accessed by contacting XXX. All the relevant data from this study are also available from the corresponding authors upon request. |
|  | **Only extra data (or data we don’t know how they can be used for) are available upon request**   - Whenever some data are listed/shared also after statements of the type (All relevant data available upon request) - All relevant data are within the paper. Additional data available upon request - All necessary data are available in the manuscript and SI Appendix. Additional raw data are available upon request - Additional patients’ parameters can be made available upon request to the corresponding author. - Additional supporting data can be made available upon request to the corresponding author - Additional material including XXXX and XXXX are available upon request. - The data available as supplementary files and if additional information is required, it is possible to send more details. - All substantial data are available in the text and the Supplementary Figures. Certificates of XXX and XXXX, XXXX data sheets, and detailed XXXX are available upon request to the corresponding author. - Information about all other data and reagents described under results will be made freely available upon request. - We declare that we have provided all the data, but the primary data will be available upon request. - Data for XXX are available upon request to the authors. Data for XXXX relevant to the analyses described are available on our XXX page - the XXX that support the findings of this study are available from the corresponding author upon reasonable request while the data are available in supplementary material. - The algorithm is available upon request - Sequencies/ Whole-brain test imaging data available upon request - The dataset employed to (optimize Eq. XX, produce figure XXX) is available upon request - Additional material including strains and scripts are available upon request. - Detailed information about the reported XX and/or the reported XX is available upon request. - They talk about material and not data - The entire bioinformatics pipeline and XX tables are available upon request. - Data files generated and analyzed during the current study, but not included as supplementary information files, are available from the corresponding author on request - Extra information on methods, data and protocols are available upon request - The XXX data for the fluids used in this study are reported in the Supplementary Tables, and the seismic data may be requested from the corresponding author - Experimental materials used to generate the results reported in this manuscript are available upon request. |
|  | **Data is deposited somewhere OR will be deposited somewhere:**   - All XXX data have been deposited in the (XX) database under accession number XXXX. XXX are available upon request. - The details of analyses used in the current study are available from the first author or corresponding author upon request. - All source data will be deposited on an institutional data repository and made available upon request to the senior author. - Authors agree to make all primary data available upon request and will upload the data on a public repository post-publication. |
|  | **Ethical privacy issues/ bounded to collaboration**   - Data availability conditional on IRB approval - The datasets generated from XXXX are available from the corresponding author on reasonable request. The De-identified XX XXXX data may be available upon request in case a mutually agreed research plan and ethical approval documents are provided. - The authors declare that all the data related to this manuscript will be made available upon request under collaborative agreement. |
|  | - The data that support the findings of this study are available, but restrictions apply to the availability of these data - When we don’t know the nature of the data: e.g., “XXXX sequences are available upon request”. And no other statement about data is present. - The full set of raw data is not currently publicly available due to ethical restrictions. A subset of the sample can be made available upon request. - Source data including raw and processed image files are available upon request from the corresponding authors - The data might be made available upon request, and some restrictions will apply. |

**Notes.** Only positive data availability statements were included in the sample. C.A. and M.W.N. manually checked and coded the data availability section of each paper. The codebook was first tested by coding 10% of the sample. The pilot phase was repeated, and the codebook was further adjusted, until intercoding reliability measures reached a satisfactory level (Kappa coefficient ≥ 0.8).

**Table S2** University affiliations (Institution status) comparison across rankings

|  | **THE** | **Shanghai (National)** | **Shanghai (ARWU)** | **QS** | **LEIDEN (PP-top 10%)** |
| --- | --- | --- | --- | --- | --- |
|  | **University (LOW) China** | | | | |
| Chongqing University | 601-800 | 20-35 | 201-300 | 751-800 | 10.4% (nr. 424) |
|  | **University (HIGH) China** | | | | |
| Zhejiang University | 94 | 3 | 52 | 53 | 11.2% (nr. 311) |
|  | **University (LOW) US** | | | | |
| Baylor University | 801-1000 | 153-158 | 601-700 | 801-1000 | 10.2% (nr. 454) |
|  | **University (HIGH) US** | | | | |
| Carnegie Mellon University | 28 | 38-39 | 97 | 51 | 14.6% (nr. 77) |

Note: All rankings are from 2021. THE=Times Higher Education World University Ranking. Shanghai (National) = Shanghai Country ranking. (ARWU)=Shanghai Academic Ranking of World Universities. QS=QS World University Ranking. Leiden (PP-top 10%) = Proportion of publication in top-10% globally.

**Table S3** Variable specifications.

| **Variables** | **Specification** | **Measurement Type** |
| --- | --- | --- |
| **Outcome variable** | | |
| Sharing data | Measures whether respondents shared their data or indicated willingness to share their data (1), or whether the respondents did not share data or indicated no willingness to share their data (0). | Dichotomous (0,1) |
| **Main predictors** | | |
| Country | Country is a dichotomous variable specifying the country of residence of the fictitious data requestor (US=0, China=1). | Dichotomous (0,1) |
| Institution | Institution is a dichotomous variable specifying whether the fictitious data requestor is affiliated with a high-ranked or lower ranked institution. (higher ranked=0, lower ranked=1). | Dichotomous (0,1) |
| Gender | Gender is a dichotomous variable specifying whether the fictitious data requestor has a feminine coded or masculine coded first name (masculine coded=0, feminine coded=1). | Dichotomous (0,1) |
| Ethnicity | Ethnicity is a dichotomous variable specifying whether the fictitious data requestor has a Chinese or typical Anglo-Saxon name (Anglo-Saxon name=0, Chinese name=1). | Dichotomous (0,1) |
| **Controls** | | |
| Physical sciences and engineering | Physical sciences and engineering=1, Other=0 | Dichotomous (0,1) |
| Life and earth sciences | Life and earth sciences=1, Other=0 | Dichotomous (0,1) |
| Biomedical and health sciences | Biomedical and health sciences=1, Other=0 | Dichotomous (0,1) |
| Social sciences and humanities | Social sciences and humanities=1, Other=0 | Dichotomous (0,1) |
| Publisher | Publisher is a categorical variable specifying the publisher where a given paper was published (Nature-branded journal =0, PNAS =1). | Dichotomous (0,1) |

**Table. S4** Codebook for inferring data-sharing willingness. (Respondents did not share data nor indicate willingness to share data =0, respondents have shared their data or indicated willingness to share data =1).

**Table S5** Response rate first and second round of emails (initial contact and reminder)

| **TREATMENT** | **RESPONSE**  **RATE**  **ROUND 1** | **RESPONSE RATE**  **ROUND 2** |
| --- | --- | --- |
| 张嘉实 (Jiashi Zhang) | 29.7% | 35.1% |
| 邢 雅丹 (Yadan Xing) | 34.3% | 35.5% |
| Hilary Witmer | 24.4% | 38.5% |
| Jeffrey Killion | 33.6% | 40.6% |

**Table S6** LPMs presented in Figure 1 (Panel A).

| **Linear probability models - Response (opened emails)** | | | |  |
| --- | --- | --- | --- | --- |
|  | **Response** | | |  |
| *Predictors* | *Estimates* |  | *CI 95%* | *CI 99%* |
| Intercept | 0.82 |  | 0.59 – 1.04 | 0.52 – 1.12 |
| Gender | -0.01 |  | -0.07 – 0.05 | -0.09 – 0.07 |
| Ethnicity | -0.07 |  | -0.13 – -0.01 | -0.15 – 0.01 |
| Uni.status | -0.01 |  | -0.07 – 0.05 | -0.09 – 0.07 |
| Biomedical and health sciences | -0.04 |  | -0.26 – 0.19 | -0.33 – 0.26 |
| Life and earth sciences | 0.07 |  | -0.17 – 0.30 | -0.24 – 0.37 |
| Physical sciences and engineering | 0.00 |  | -0.23 – 0.23 | -0.30 – 0.30 |
| Publisher | 0.00 |  | -0.14 – 0.14 | -0.18 – 0.19 |
| Observations | 770 | |  |  |
| R^2^ / R^2^ adjusted | 0.015 / 0.006 | |  |  |
| AIC | 879.152 | |  |  |

**Table S7** LPMs presented in Figure 1 (Panel B).

| **Linear probability models - Willingness to share (opened emails)** | | | |  |
| --- | --- | --- | --- | --- |
|  | **Willingness** | | |  |
| *Predictors* | *Estimates* |  | *CI 95%* | *CI 99%* |
| Intercept | 0.38 |  | 0.12 – 0.64 | 0.03 – 0.72 |
| Gender | -0.02 |  | -0.07 – 0.05 | -0.10 – 0.06 |
| Ethnicity | -0.01 |  | -0.08 – 0.04 | -0.09 – 0.07 |
| Uni.status | 0.05 |  | -0.01 – 0.11 | -0.02 – 0.13 |
| Biomedical and health sciences | -0.18 |  | -0.44 – 0.08 | -0.52 – 0.16 |
| Life and earth sciences | -0.06 |  | -0.33 – 0.21 | -0.41 – 0.30 |
| Physical sciences and engineering | -0.16 |  | -0.42 – 0.11 | -0.50 – 0.19 |
| Publisher | -0.00 |  | -0.14 – 0.13 | -0.18 – 0.17 |
| Observations | 770 | |  |  |
| R^2^ / R^2^ adjusted | 0.018 / 0.009 | |  |  |
| AIC | 865.198 | |  |  |

**Table S8** LPMs presented in Figure S4 (Panel A).

| **Linear probability models - Response (full sample)** | | | |  |
| --- | --- | --- | --- | --- |
|  | **Response** | | |  |
| *Predictors* | *Estimates* |  | *CI 95%* | *CI 99%* |
| Intercept | 0.57 |  | 0.34 – 0.81 | 0.27 – 0.88 |
| Gender | -0.03 |  | -0.09 – 0.03 | -0.11 – 0.05 |
| Ethnicity | -0.05 |  | -0.11 – 0.01 | -0.13 – 0.03 |
| Uni.status | -0.02 |  | -0.08 – 0.04 | -0.09 – 0.06 |
| Biomedical and health sciences | -0.03 |  | -0.26 – 0.20 | -0.33 – 0.27 |
| Life and earth sciences | 0.11 |  | -0.13 – 0.34 | -0.21 – 0.42 |
| Physical sciences and engineering | 0.03 |  | -0.20 – 0.27 | -0.27 – 0.34 |
| Publisher | 0.16 |  | 0.02 – 0.30 | -0.03 – 0.35 |
| Observations | 1092 | |  |  |
| R^2^ / R^2^ adjusted | 0.017 / 0.010 | |  |  |
| AIC | 1578.762 | |  |  |

**Table S9** LPMs presented in Figure S4 (Panel B).

| **Linear probability models - Willingness to share (full sample)** | | | |  |
| --- | --- | --- | --- | --- |
|  | **Willingness** | | |  |
| *Predictors* | *Estimates* |  | *CI 95%* | *CI 99%* |
| Intercept | 0.26 |  | 0.06 – 0.46 | -0.00 – 0.52 |
| Gender | -0.02 |  | -0.06 – 0.03 | -0.07 – 0.04 |
| Ethnicity | -0.01 |  | -0.06 – 0.03 | -0.07 – 0.04 |
| Uni.status | 0.03 |  | -0.01 – 0.08 | -0.02 – 0.09 |
| Biomedical and health sciences | -0.12 |  | -0.32 – 0.07 | -0.38 – 0.14 |
| Life and earth sciences | -0.01 |  | -0.22 – 0.19 | -0.29 – 0.26 |
| Physical sciences and engineering | -0.09 |  | -0.30 – 0.11 | -0.36 – 0.17 |
| Publisher | 0.04 |  | -0.08 – 0.16 | -0.12 – 0.20 |
| Observations | 1092 | |  |  |
| R^2^ / R^2^ adjusted | 0.015 / 0.009 | |  |  |
| AIC | 935.195 | |  |  |

**Table S10** LPMs presented in Figure 2 (Panel A).

| **Linear probability models - Response (opened emails)** | | | |  |
| --- | --- | --- | --- | --- |
|  | **Response** | | |  |
| *Predictors* | *Estimates* |  | *CI 95%* | *CI 99%* |
| Intercept | 0.49 |  | 0.22 – 0.76 | 0.13 – 0.84 |
| Gender | 0.02 |  | -0.05 – 0.07 | -0.06 – 0.10 |
| Country | 0.00 |  | -0.05 – 0.08 | -0.08 – 0.08 |
| Uni.status | 0.01 |  | -0.06 – 0.06 | -0.07 – 0.09 |
| Biomedical and health sciences | 0.21 |  | -0.06 – 0.48 | -0.14 – 0.56 |
| Life and earth sciences | 0.25 |  | -0.03 – 0.52 | -0.11 – 0.61 |
| Physical sciences and engineering | 0.27 |  | -0.00 – 0.54 | -0.09 – 0.62 |
| Publisher | -0.03 |  | -0.17 – 0.11 | -0.21 – 0.15 |
| Observations | 802 | |  |  |
| R^2^ / R^2^ adjusted | 0.008 / -0.001 | |  |  |
| AIC | 990.691 | |  |  |

**Table S11** LPMs presented in Figure 2 (Panel B).

| **Linear probability models - Willingness to share (opened emails)** | | | |  |
| --- | --- | --- | --- | --- |
|  | **Willingness** | | |  |
| *Predictors* | *Estimates* |  | *CI 95%* | *CI 99%* |
| Intercept | 0.13 |  | -0.06 – 0.31 | -0.12 – 0.37 |
| Gender | 0.05 |  | -0.01 – 0.11 | -0.02 – 0.13 |
| Country | -0.01 |  | -0.07 – 0.05 | -0.09 – 0.06 |
| Uni.status | -0.00 |  | -0.06 – 0.06 | -0.08 – 0.07 |
| Biomedical and health sciences | 0.04 |  | -0.14 – 0.23 | -0.20 – 0.28 |
| Life and earth sciences | 0.16 |  | -0.04 – 0.36 | -0.10 – 0.42 |
| Physical sciences and engineering | 0.12 |  | -0.07 – 0.31 | -0.13 – 0.37 |
| Publisher | -0.14 |  | -0.23 – -0.06 | -0.25 – -0.04 |
| Observations | 802 | |  |  |
| R^2^ / R^2^ adjusted | 0.026 / 0.017 | |  |  |
| AIC | 851.185 | |  |  |

**Table S12** LPMs presented in Figure S5 (Panel A).

| **Linear probability models - Response (opened emails)** | | | |  |
| --- | --- | --- | --- | --- |
|  | **Response** | | |  |
| *Predictors* | *Estimates* |  | *CI 95%* | *CI 99%* |
| Intercept | 0.35 |  | 0.12 – 0.59 | 0.05 – 0.66 |
| Gender | 0.01 |  | -0.05 – 0.07 | -0.07 – 0.09 |
| Country | 0.03 |  | -0.02 – 0.09 | -0.04 – 0.11 |
| Uni.status | 0.02 |  | -0.04 – 0.07 | -0.06 – 0.09 |
| Biomedical and health sciences | 0.11 |  | -0.12 – 0.33 | -0.19 – 0.41 |
| Life and earth sciences | 0.22 |  | -0.02 – 0.46 | -0.10 – 0.53 |
| Physical sciences and engineering | 0.21 |  | -0.02 – 0.44 | -0.10 – 0.52 |
| Publisher | 0.07 |  | -0.07 – 0.20 | -0.11 – 0.25 |
| Observations | 1097 | |  |  |
| R^2^ / R^2^ adjusted | 0.014 / 0.008 | |  |  |
| AIC | 1590.114 | |  |  |

**Table S13** LPMs presented in Figure S5 (Panel B).

| **Linear probability models - Willingness to share (opened emails)** | | | |  |
| --- | --- | --- | --- | --- |
|  | **Willingness** | | |  |
| *Predictors* | *Estimates* |  | *CI 95%* | *CI 99%* |
| Intercept | 0.09 |  | -0.06 – 0.24 | -0.10 – 0.28 |
| Gender | 0.04 |  | -0.01 – 0.08 | -0.02 – 0.09 |
| Country | 0.00 |  | -0.04 – 0.05 | -0.05 – 0.06 |
| Uni.status | 0.00 |  | -0.04 – 0.05 | -0.05 – 0.06 |
| Biomedical and health sciences | 0.02 |  | -0.13 – 0.16 | -0.17 – 0.21 |
| Life and earth sciences | 0.14 |  | -0.02 – 0.29 | -0.07 – 0.34 |
| Physical sciences and engineering | 0.09 |  | -0.06 – 0.24 | -0.11 – 0.29 |
| Publisher | -0.10 |  | -0.17 – -0.03 | -0.19 – -0.01 |
| Observations | 1097 | |  |  |
| R^2^ / R^2^ adjusted | 0.023 / 0.017 | |  |  |
| AIC | 896.234 | |  |  |

**Table S14** LPMs presented in Figure 3 (Panel A).

| **Linear probability models - Response (opened emails)** | | | |  |
| --- | --- | --- | --- | --- |
|  | **Response** | | |  |
| *Predictors* | *Estimates* |  | *CI 95%* | *CI 99%* |
| Intercept | 0.70 |  | 0.49 – 0.91 | 0.43 – 0.98 |
| Gender | -0.00 |  | -0.05 – 0.05 | -0.07 – 0.06 |
| Ethnicity | -0.07 |  | -0.12 – -0.02 | -0.14 – -0.00 |
| Uni.status | 0.00 |  | -0.05 – 0.05 | -0.06 – 0.07 |
| Biomedical and health sciences | 0.08 |  | -0.12 – 0.29 | -0.19 – 0.35 |
| Life and earth sciences | 0.14 |  | -0.08 – 0.35 | -0.14 – 0.42 |
| Physical sciences and engineering | 0.12 |  | -0.09 – 0.33 | -0.16 – 0.39 |
| Publisher | -0.02 |  | -0.13 – 0.09 | -0.17 – 0.13 |
| Observations | 1179 | |  |  |
| R^2^ / R^2^ adjusted | 0.009 / 0.003 | |  |  |
| AIC | 1379.746 | |  |  |

**Table S15** LPMs presented in Figure S6.

| **Linear probability models - Response (full sample)** | | | |  |
| --- | --- | --- | --- | --- |
|  | **Response** | | |  |
| *Predictors* | *Estimates* |  | *CI 95%* | *CI 99%* |
| Intercept | 0.49 |  | 0.30 – 0.68 | 0.24 – 0.74 |
| Gender | -0.02 |  | -0.07 – 0.03 | -0.08 – 0.04 |
| Ethnicity | -0.03 |  | -0.08 – 0.02 | -0.10 – 0.04 |
| Uni.status | -0.00 |  | -0.05 – 0.05 | -0.06 – 0.06 |
| Biomedical and health sciences | 0.04 |  | -0.15 – 0.23 | -0.20 – 0.28 |
| Life and earth sciences | 0.16 |  | -0.04 – 0.35 | -0.10 – 0.41 |
| Physical sciences and engineering | 0.12 |  | -0.07 – 0.31 | -0.13 – 0.37 |
| Publisher | 0.10 |  | -0.01 – 0.21 | -0.05 – 0.25 |
| Observations | 1634 | |  |  |
| R^2^ / R^2^ adjusted | 0.012 / 0.008 | |  |  |
| AIC | 2359.327 | |  |  |

**Table S16** Logistic regression model corresponding to LPMs presented in Figure 1 (Panel A).

| **Logistic Regression Model - Response (opened emails)** | | |  |  |
| --- | --- | --- | --- | --- |
|  | **Response** | | |  |
| *Predictors* | *Odds Ratios* |  | *CI 95%* | *CI 99%* |
| Intercept | 4.40 |  | 1.29 – 20.24 | 0.89 – 36.68 |
| Ethnicity | 0.66 |  | 0.47 – 0.93 | 0.42 – 1.03 |
| Gender | 0.96 |  | 0.69 – 1.34 | 0.62 – 1.49 |
| Uni.status | 0.95 |  | 0.68 – 1.33 | 0.61 – 1.48 |
| Publisher | 1.01 |  | 0.48 – 2.33 | 0.39 – 3.11 |
| Biomedical and health sciences | 0.82 |  | 0.18 – 2.76 | 0.10 – 3.95 |
| Life and earth sciences | 1.53 |  | 0.32 – 5.55 | 0.18 – 8.18 |
| Physical sciences and engineering | 1.01 |  | 0.22 – 3.49 | 0.12 – 5.04 |
| Observations | 770 | |  |  |
| R^2^ Tjur | 0.015 | |  |  |
| AIC | 850.999 | |  |  |

**Table S17** Logistic regression model corresponding to LPMs presented in Figure 1 (Panel B).

| **Logistic Regression Model - Willingness to share (opened emails)** | | |  |  |
| --- | --- | --- | --- | --- |
|  | **Willingness** | | |  |
| *Predictors* | *Odds Ratios* |  | *CI 95%* | *CI 99%* |
| Intercept | 0.60 |  | 0.17 – 1.87 | 0.11 – 2.69 |
| Ethnicity | 0.89 |  | 0.63 – 1.24 | 0.57 – 1.38 |
| Gender | 0.94 |  | 0.67 – 1.32 | 0.60 – 1.46 |
| Uni.status | 1.36 |  | 0.97 – 1.91 | 0.87 – 2.13 |
| Publisher | 0.98 |  | 0.41 – 2.10 | 0.30 – 2.63 |
| Biomedical and health sciences | 0.40 |  | 0.13 – 1.36 | 0.09 – 2.10 |
| Life and earth sciences | 0.77 |  | 0.24 – 2.71 | 0.17 – 4.21 |
| Physical sciences and engineering | 0.47 |  | 0.15 – 1.65 | 0.10 – 2.55 |
| Observations | 770 | |  |  |
| R^2^ Tjur | 0.018 | |  |  |
| AIC | 840.271 | |  |  |

**Table S18** Logistic regression model corresponding to LPMs presented in Figure S4 (Panel A).

| **Logistic Regression Model - Response (opened emails)** | | |  |  |
| --- | --- | --- | --- | --- |
|  | **Response** | | |  |
| *Predictors* | *Odds Ratios* |  | *CI 95%* | *CI 99 %* |
| Intercept | 1.35 |  | 0.53 – 3.50 | 0.39 – 4.79 |
| Ethnicity | 0.82 |  | 0.65 – 1.05 | 0.60 – 1.13 |
| Gender | 0.88 |  | 0.69 – 1.12 | 0.64 – 1.21 |
| Uni.status | 0.93 |  | 0.73 – 1.18 | 0.68 – 1.28 |
| Publisher | 1.98 |  | 1.03 – 4.00 | 0.84 – 5.08 |
| Biomedical and health sciences | 0.88 |  | 0.35 – 2.22 | 0.25 – 3.01 |
| Life and earth sciences | 1.55 |  | 0.58 – 4.07 | 0.42 – 5.57 |
| Physical sciences and engineering | 1.15 |  | 0.44 – 2.94 | 0.32 – 4.00 |
| Observations | 1092 | |  |  |
| R^2^ Tjur | 0.017 | |  |  |
| AIC | 1505.344 | |  |  |

**Table S19** Logistic regression model corresponding to LPMs presented in Figure S4 (Panel B).

| **Logistic Regression Model - Willingness to share (opened emails)** | | |  |  |
| --- | --- | --- | --- | --- |
|  | **Willingness** | | |  |
| *Predictors* | *Odds Ratios* |  | *CI 95%* | *CI 99 %* |
| Intercept | 0.35 |  | 0.11 – 0.95 | 0.07 – 1.28 |
| Ethnicity | 0.91 |  | 0.66 – 1.26 | 0.60 – 1.39 |
| Gender | 0.89 |  | 0.64 – 1.23 | 0.58 – 1.36 |
| Uni.status | 1.28 |  | 0.93 – 1.77 | 0.84 – 1.97 |
| Publisher | 1.36 |  | 0.57 – 2.87 | 0.42 – 3.57 |
| Biomedical and health sciences | 0.45 |  | 0.17 – 1.42 | 0.12 – 2.16 |
| Life and earth sciences | 0.93 |  | 0.33 – 3.02 | 0.24 – 4.63 |
| Physical sciences and engineering | 0.57 |  | 0.20 – 1.84 | 0.15 – 2.81 |
| Observations | 1092 | |  |  |
| R^2^ Tjur | 0.015 | |  |  |
| AIC | 978.138 | |  |  |

**Table S20** Logistic regression model corresponding to LPMs presented in Figure 3.

| **Logistic Regression Model - Response (opened emails)** | | |  |  |
| --- | --- | --- | --- | --- |
|  | **Response** | | |  |
| *Predictors* | *Odds Ratios* |  | *CI 95 %* | *CI 99%* |
| Intercept | 2.49 |  | 0.97 – 6.93 | 0.72 – 9.88 |
| Ethnicity | 0.67 |  | 0.50 – 0.90 | 0.45 – 0.98 |
| Gender | 0.98 |  | 0.75 – 1.28 | 0.69 – 1.39 |
| Uni.status | 1.02 |  | 0.78 – 1.32 | 0.72 – 1.44 |
| Publisher | 0.90 |  | 0.52 – 1.62 | 0.44 – 1.97 |
| Biomedical and health sciences | 1.48 |  | 0.55 – 3.69 | 0.39 – 4.90 |
| Life and earth sciences | 2.00 |  | 0.71 – 5.24 | 0.49 – 7.08 |
| Physical sciences and engineering | 1.77 |  | 0.64 – 4.52 | 0.45 – 6.05 |
| Observations | 1179 | |  |  |
| R^2^ Tjur | 0.010 | |  |  |
| AIC | 1331.248 | |  |  |

**Table S21** Logistic regression model corresponding to LPMs presented in Figure S6.

| **Logistic Regression Model - Response (opened emails)** | | |  |  |
| --- | --- | --- | --- | --- |
|  | **Response** | | |  |
| *Predictors* | *Odds Ratios* |  | *CI 95%* | *CI 99 %* |
| Intercept | 0.42 |  | 0.14 – 1.09 | 0.10 – 1.46 |
| Ethnicity | 0.85 |  | 0.64 – 1.14 | 0.58 – 1.25 |
| Gender | 1.11 |  | 0.85 – 1.47 | 0.78 – 1.60 |
| Uni.status | 1.23 |  | 0.93 – 1.63 | 0.86 – 1.77 |
| Publisher | 0.67 |  | 0.31 – 1.28 | 0.24 – 1.54 |
| Biomedical and health sciences | 0.55 |  | 0.22 – 1.59 | 0.16 – 2.32 |
| Life and earth sciences | 1.08 |  | 0.41 – 3.18 | 0.30 – 4.66 |
| Physical sciences and engineering | 0.74 |  | 0.28 – 2.16 | 0.21 – 3.16 |
| Observations | 1179 | |  |  |
| R^2^ Tjur | 0.017 | |  |  |
| AIC | 1255.649 | |  |  |

**Table S22** LPMs presented in Figure 4 (Panel A).

| **Linear Regression Model - Response (opened emails)** | | |  |  |
| --- | --- | --- | --- | --- |
|  | **Response** | | |  |
| *Predictors* | *Estimates* |  | *CI 95%* | *CI 99%* |
| Intercept | 0.72 |  | 0.51 – 0.93 | 0.44 – 1.00 |
| Chinese Male | -0.10 |  | -0.17 – -0.03 | -0.19 – -0.01 |
| Uni.status | 0.00 |  | -0.05 – 0.05 | -0.06 – 0.07 |
| Publisher | -0.02 |  | -0.13 – 0.09 | -0.17 – 0.13 |
| Biomedical and health sciences | 0.08 |  | -0.12 – 0.29 | -0.19 – 0.36 |
| Life and earth sciences | 0.14 |  | -0.07 – 0.35 | -0.14 – 0.42 |
| Physical sciences and engineering | 0.12 |  | -0.09 – 0.33 | -0.16 – 0.39 |
| Anglo-Saxon Female | -0.04 |  | -0.12 – 0.04 | -0.15 – 0.07 |
| Chinese Female | 0.02 |  | -0.05 – 0.08 | -0.07 – 0.10 |
| Observations | 1179 | |  |  |
| R^2^ / R^2^ adjusted | 0.010 / 0.004 | |  |  |
| AIC | 1380.634 | |  |  |

**Table S23** LPMs presented in Figure 4 (Panel B).

| **Linear Regression Model - Willingness (opened emails)** | | |  |  |
| --- | --- | --- | --- | --- |
|  | **Willingness** | | |  |
| *Predictors* | *Estimates* |  | *CI 95%* | *CI 95%* |
| Intercept | 0.32 |  | 0.12 – 0.53 | 0.06 – 0.59 |
| Chinese Male | -0.07 |  | -0.15 – -0.00 | -0.17 – 0.02 |
| Uni.status | 0.03 |  | -0.01 – 0.08 | -0.03 – 0.10 |
| Publisher | -0.06 |  | -0.15 – 0.03 | -0.18 – 0.06 |
| Biomedical and health sciences | -0.11 |  | -0.30 – 0.09 | -0.36 – 0.15 |
| Life and earth sciences | 0.02 |  | -0.19 – 0.22 | -0.25 – 0.29 |
| Physical sciences and engineering | -0.06 |  | -0.26 – 0.14 | -0.32 – 0.21 |
| Anglo-Saxon Female | -0.05 |  | -0.13 – 0.04 | -0.16 – 0.07 |
| Chinese Female | 0.05 |  | -0.01 – 0.10 | -0.03 – 0.12 |
| Observations | 1179 | |  |  |
| R^2^ / R^2^ adjusted | 0.019 / 0.013 | |  |  |
| AIC | 1285.842 | |  |  |

**Table S24** LPMs presented in Figure S7 (Panel A).

|  | **Response** | | |  |
| --- | --- | --- | --- | --- |
| *Predictors* | *Estimates* |  | *CI 95%* | *CI 99%* |
| Intercept | 0.52 |  | 0.33 – 0.71 | 0.27 – 0.77 |
| Chinese Male | -0.08 |  | -0.15 – -0.00 | -0.17 – 0.02 |
| Uni.status | -0.00 |  | -0.05 – 0.05 | -0.06 – 0.06 |
| Publisher | 0.10 |  | -0.01 – 0.21 | -0.05 – 0.25 |
| Biomedical and health sciences | 0.04 |  | -0.14 – 0.23 | -0.20 – 0.29 |
| Life and earth sciences | 0.16 |  | -0.03 – 0.35 | -0.09 – 0.41 |
| Physical sciences and engineering | 0.12 |  | -0.07 – 0.31 | -0.13 – 0.37 |
| Anglo-Saxon Female | -0.08 |  | -0.16 – 0.00 | -0.19 – 0.03 |
| Chinese Female | 0.01 |  | -0.05 – 0.07 | -0.07 – 0.09 |
| Observations | 1634 | |  |  |
| R^2^ / R^2^ adjusted | 0.014 / 0.009 | |  |  |
| AIC | 2358.380 | |  |  |

**Table S25** LPMs presented in Figure S7 (Panel B).

| **Linear Regression Model - Willingness (full sample)** | | |  |  |
| --- | --- | --- | --- | --- |
|  | **Willingness** | | |  |
| *Predictors* | *Estimates* |  | *CI 95 %* | *CI 99%* |
| Intercept | 0.23 |  | 0.08 – 0.39 | 0.03 – 0.44 |
| Chinese Male | -0.06 |  | -0.11 – 0.00 | -0.13 – 0.02 |
| Uni.status | 0.02 |  | -0.01 – 0.06 | -0.02 – 0.07 |
| Publisher | -0.02 |  | -0.10 – 0.06 | -0.13 – 0.08 |
| Biomedical and health sciences | -0.08 |  | -0.23 – 0.07 | -0.28 – 0.12 |
| Life and earth sciences | 0.04 |  | -0.12 – 0.20 | -0.17 – 0.25 |
| Physical sciences and engineering | -0.03 |  | -0.18 – 0.13 | -0.23 – 0.18 |
| Anglo-Saxon Female | -0.05 |  | -0.11 – 0.01 | -0.13 – 0.03 |
| Chinese Female | 0.04 |  | -0.01 – 0.08 | -0.02 – 0.09 |
| Observations | 1634 | |  |  |
| R^2^ / R^2^ adjusted | 0.018 / 0.013 | |  |  |
| AIC | 1371.325 | |  |  |

**Table S26** Geographical distribution of participants.

| **Country of affiliation respondents** | **N** |
| --- | --- |
| NORTH AMERICA (EXCL. MEXICO) | 633 |
| EUROPE* | 653 |
| REMAINING COUNTRIES | 348 |

Note. *Europe = Germany, United Kingdom, France, Italy, Netherlands, Switzerland, Spain, Sweden, Austria, Portugal, Denmark, Belgium, Norway, Finland, Greece, Hungary, Ireland, Poland, Czech Republic, Estonia, Luxembourg, Slovakia, Lithuania, Slovenia
